# Net Primary Productivity Retrieval Based on ESTARFM Fusion and an Improved CASA Model

**DOI:** 10.3390/plants15101436

**Published:** 2026-05-08

**Authors:** Yuanji Cai, Chunling Chen, Wanning Li, Hao Han, Zhichao Ren, Zihao Wang, Ziyi Feng

**Affiliations:** 1 College of Information and Electrical Engineering, Shenyang Agricultural University, Shenyang 110866, China; 2024240160@stu.syau.edu.cn (Y.C.); chenchunling@syau.edu.cn (C.C.); 2023240092@stu.syau.edu.cn (W.L.); 2022200007@stu.syau.edu.cn (H.H.); 2025240104@stu.syau.edu.cn (Z.R.); 2025240106@stu.syau.edu.cn (Z.W.); 2 Liaoning Provincial Key Laboratory of Smart Agriculture Technology, Shenyang 110866, China; 3 High-Resolution Earth Observation System, Liaoning Forest and Grass Resources and Environment Remote Sensing Research and Application Center, Shenyang 110866, China

**Keywords:** maize, rice, net primary productivity, improved CASA model, remote sensing, spatiotemporal fusion

## Abstract

Net primary productivity (NPP) is an important indicator of ecosystem carbon accumulation capacity and vegetation productivity potential, and its accurate estimation is of great significance for agricultural management and regional carbon cycle research. To address the problem that the temporal continuity of single-source optical remote sensing data is easily affected by cloud cover, this study used Sentinel-2 imagery and the Moderate Resolution Imaging Spectroradiometer (MODIS) Normalized Difference Vegetation Index (NDVI) product as data sources and constructed an NDVI time series with high spatial and temporal resolution for the study area based on the Enhanced Spatial and Temporal Adaptive Reflectance Fusion Model (ESTARFM) method. On this basis, the Simple Ratio (SR) index was incorporated to supplement canopy information, and the key parameters of the Carnegie–Ames–Stanford Approach (CASA) model were differentially optimized for different crop types, thereby enabling remote sensing-based estimation of crop NPP. The results showed that the fused NDVI effectively compensated for observation gaps caused by cloud interference, and its temporal variation was generally consistent with the crop growth process. In addition, the Fraction of Photosynthetically Active Radiation (FPAR) improved with the fused NDVI, which effectively characterized phenological differences among crops. Compared with the unoptimized model, the improved model significantly improved NPP estimation accuracy for both maize and rice. Specifically, for maize, the coefficient of determination ($R^{2}$) increased from 0.75 to 0.88, and the mean absolute percentage error (MAPE) decreased from 67.00% to 34.68%. For rice, the MAPE decreased from 78.51% to 23.43%, while the mean absolute error (MAE) decreased from 345.1 $\mathrm{gC}\cdot m^{-2}\cdot a^{-1}$ to 95.6 $\mathrm{gC}\cdot m^{-2}\cdot a^{-1}$. These results indicate that constructing a highly continuous vegetation index time series through spatiotemporal fusion, together with optimizing the CASA model by incorporating the SR index and crop-specific parameterization, can effectively improve the stability and accuracy of NPP estimation for agricultural crops.

## 1. Introduction

Vegetation NPP influences the carbon balance of terrestrial ecosystems and is one of the core indicators used to characterize the carbon sequestration capacity and growth status of terrestrial vegetation [1,2,3]. In agroecosystems, the spatiotemporal variation in crop NPP is influenced not only by meteorological factors such as radiation, temperature, and water availability, but also by crop type and field management practices [4,5]. Therefore, obtaining continuous and reliable NPP information at the regional scale provides an important basis for comparing crop carbon sequestration capacity and investigating its driving mechanisms [6].

Traditional approaches to NPP estimation mainly rely on large-scale harvesting methods. However, these methods cause substantial disturbance to vegetation, are labor-intensive to implement, and require considerable time and manpower. With the development of satellite remote sensing technology, remote sensing-based estimation of crop NPP has become an important approach [7]. Existing methods for NPP estimation can be broadly classified into three categories: statistical models, process-based models, and light-use efficiency models. Statistical models are typically based on field observations and estimate NPP by establishing empirical relationships between NPP and climatic factors such as temperature and precipitation. This approach is relatively simple to implement and requires easily accessible parameters; however, it does not adequately account for non-climatic influences, such as differences in vegetation type and management practices, and therefore cannot accurately capture variations in NPP [8]. Process-based models are founded on plant physiological processes and estimate NPP by simulating mechanisms such as photosynthesis, respiration, and carbon allocation. Although these models have strong mechanistic interpretability, their application over large areas is constrained by the large number of model parameters and the high-quality requirements for input data [9,10]. In contrast, light-use efficiency models estimate NPP based on absorbed photosynthetically active radiation and vegetation light-use efficiency, while incorporating climatic regulators such as temperature and precipitation. Compared with process-based models, light-use efficiency models require parameters that are easier to obtain and, at the same time, retain a physiological basis for vegetation functioning [11,12].

Among the various light-use efficiency models, the CASA is one of the most widely used. Previous studies have shown that the simulation results of the CASA model are highly consistent with the MODIS NPP product [13]. In addition, most of the parameters required by the CASA model can be derived from remote sensing imagery, which, to some extent, compensates for the limitations arising from insufficient ground-based observations. Wang et al. [14] used the CASA model to simulate the NPP of summer maize in Shanxi Province, thereby demonstrating its application potential in agroecosystems. In addition, Wang et al. [15] further integrated Sentinel-2 imagery, meteorological data, and deep-learning-based classification results to estimate forest NPP at the regional scale, indicating that the CASA model also has considerable potential for productivity simulation across different ecosystems.

It should be noted that the application of the CASA model in agroecosystems still involves uncertainties in both key parameter settings and canopy characterization. On the one hand, the maximum light-use efficiency and its related parameters are commonly assigned using fixed values or empirical schemes. However, previous studies have shown that light-use efficiency differs significantly among crop types, and neglecting such differences may reduce the accuracy of productivity simulations in agricultural ecosystems [16]. On the other hand, canopy characterization based on NDVI is prone to saturation under conditions of high vegetation cover, thereby affecting FPAR retrieval and NPP estimation. In contrast, the SR has a higher saturation threshold under dense vegetation conditions and can therefore complement canopy structural information to a certain extent [17]. Therefore, optimizing the key parameters of the CASA model for different crop types and incorporating the SR index to complement canopy information may further improve the accuracy of remote sensing-based estimation of NPP in agricultural ecosystems.

In recent years, spatiotemporal fusion techniques have been widely used to generate remote sensing data series with high spatiotemporal resolution, thereby compensating for the limitations of a single data source in either temporal or spatial resolution. Existing spatiotemporal fusion methods can generally be classified into three categories: transformation-based methods, reconstruction-based methods, and learning-based methods. Among these, reconstruction-based models represented by the Spatial and Temporal Adaptive Reflectance Fusion Model (STARFM) and ESTARFM have been widely applied in vegetation dynamics monitoring because of their clear theoretical basis and mature implementation [18,19,20]. Compared with STARFM, ESTARFM introduces mixed-pixel decomposition and similar-pixel selection mechanisms, making it more suitable for agricultural areas [19].

Previous studies have shown that spatiotemporal fusion research has mainly focused on the combined use of MODIS and Landsat data [18,19]. Compared with Landsat, Sentinel-2 provides higher spatial resolution and a shorter revisit interval, enabling more detailed characterization of vegetation dynamics in agricultural areas [21,22]. Therefore, integrating Sentinel-2 and MODIS data through spatiotemporal fusion can provide higher-spatiotemporal-resolution data support for regional-scale crop monitoring. However, during the peak growing season in July and August, the study area is frequently affected by cloudy weather, which substantially reduces the availability of optical remote sensing imagery and consequently affects the continuity and stability of the input data required by the CASA model. In agroecosystems, the peak growing season is precisely the stage during which crop canopy dynamics and carbon accumulation are most pronounced. Therefore, improving the continuity of remote sensing inputs during cloudy periods is critical for enhancing the stability of the CASA model and improving the accuracy of crop NPP estimation.

In summary, although ESTARFM, CASA, and SR-based vegetation indices have been widely applied in previous studies, their integrated application for crop-specific NPP estimation under cloudy growing-season conditions remains insufficiently explored. The novelty of this study lies not in the isolated use of these methods, but in the development of an integrated crop-specific NPP estimation framework that links high-spatiotemporal-resolution NDVI reconstruction, improved FPAR estimation, and crop-specific CASA parameterization. Specifically, Sentinel-2 and MODIS NDVI data were fused using ESTARFM to construct a temporally continuous high-spatial-resolution NDVI series during the crop growing season, thereby reducing the influence of cloud contamination on CASA model inputs. The SR index was incorporated together with NDVI to improve FPAR estimation and alleviate the saturation effect under dense crop canopies. In addition, the key parameters of the CASA model were differentially optimized for maize and rice to better represent crop-specific differences in canopy development and productivity formation. This framework aims to improve the temporal continuity, physiological interpretability, and crop-specific applicability of remote-sensing-based NPP estimation in agricultural ecosystems.

## 2. Materials and Methods

### 2.1. Overview of the Study Area

**Figure 1:**
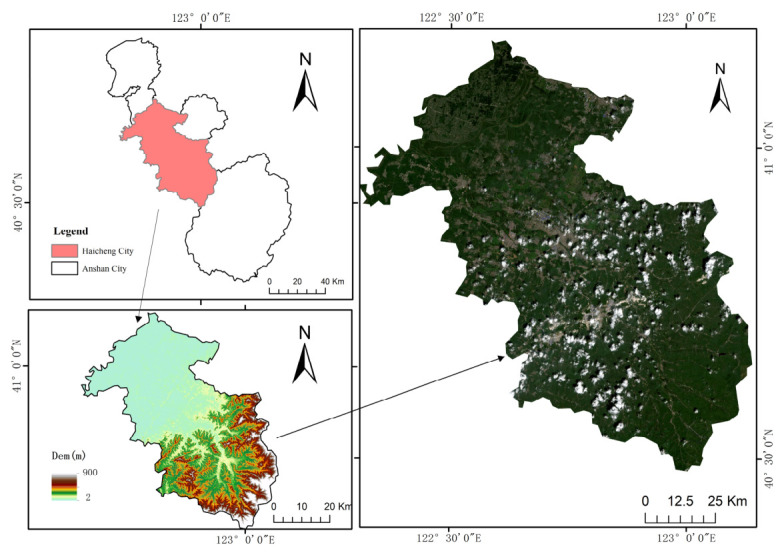
Overview of the study area.

The study area is located in Haicheng City, Liaoning Province, China, and belongs to the warm temperate monsoon climatic zone, with a mean annual temperature of 9.3 °C and a mean annual precipitation of 710.2 mm. The region is characterized by flat terrain and fertile soils, and the main crops are maize and rice under a single-cropping system [23]. The sampling area is situated at the Precision Agriculture Aviation Research Base of Shenyang Agricultural University in Haicheng City, Liaoning Province (40°58′45.39″ N, 122°43′47.01″ E; elevation: 13 m) (Figure 1). This study focuses on maize and rice and primarily analyzes the spatial distribution of net primary productivity during the growing season [24].

### 2.2. Data Collection

#### 2.2.1. Field Data Collection

To obtain accurate NPP data, field sampling and laboratory analyses were conducted from September to October 2025. Sampling points were uniformly distributed across the study area. In each experimental field, sampling points were arranged at the four corners and the center, and the mean value of the measurements from all sampling points was used to represent the sample value of that field. After field sampling, the maize and rice samples were oven-dried. First, the samples were dried at 105 °C for 1 h to rapidly terminate microbial activity, and then continuously dried at 65 °C for 48 h until a constant weight was reached. After drying, the sample’s dry weight was determined using a high-precision balance, and the samples were then crushed and ground for carbon content analysis. The product of carbon content and dry weight was used to represent the net primary productivity of each sample.

#### 2.2.2. Climate Data Collection

The meteorological data used in this study were derived from the ERA5-Land reanalysis dataset provided by the European Centre for Medium-Range Weather Forecasts (ECMWF), with a spatial resolution of 0.1°. The selected variables included 2 m air temperature, precipitation, evapotranspiration, and solar radiation. As the original unit of solar radiation was J/m^2^, whereas the CASA model requires MJ/m^2^, unit conversion was performed before model calculation. The hourly meteorological data were then aggregated to the monthly scale, and preprocessing procedures, including resampling, reprojection, and clipping to the study area, were conducted in ArcGIS Desktop 10.8 (Esri, Redlands, CA, USA) to ensure consistency between the meteorological and remote sensing datasets in terms of spatial resolution and coordinate system.

#### 2.2.3. Remote Sensing Data Collection and Preprocessing

##### MODIS Data

The NDVI data used in this study were derived from the MOD13Q1 NDVI product released by the National Aeronautics and Space Administration (NASA). This product has a temporal resolution of 16 days and a spatial resolution of 250 m, and it effectively reflects the growth status of surface vegetation. According to the requirements of this study, MOD13Q1 NDVI data covering the study area during the study period were selected as the source of time-series NDVI data. The original data were preprocessed through reprojection, mosaicking, and clipping, and a MODIS NDVI dataset covering the entire study area was ultimately generated. This dataset was mainly used for spatiotemporal fusion with Sentinel-2 NDVI data to construct an NDVI time series with high spatiotemporal resolution for the study area.

##### Satellite Remote Sensing Data

Sentinel-2 is a high-resolution Earth observation mission in a polar, sun-synchronous orbit implemented by the European Space Agency (ESA) under the Copernicus Program and is mainly used for land surface remote sensing monitoring and related application services. Sentinel-2A, Sentinel-2B, and Sentinel-2C were launched in 2015, 2017, and 2024, respectively. The Sentinel-2 constellation is equipped with a multispectral instrument that acquires imagery in 13 spectral bands spanning the visible, near-infrared (NIR), and shortwave infrared (SWIR) regions. Owing to its constellation-based operation and wide-swath imaging capability, Sentinel-2 provides a revisit interval of up to 5 days at the equator and even shorter revisit times at higher latitudes, thereby providing high-spatiotemporal-resolution data support for dynamic monitoring of agricultural vegetation [25,26]. Sentinel-2 imagery can be obtained from the Copernicus Data Space Ecosystem platform. Table 1 presents the detailed parameters of each band.

### 2.3. Methods

**Figure 2:**
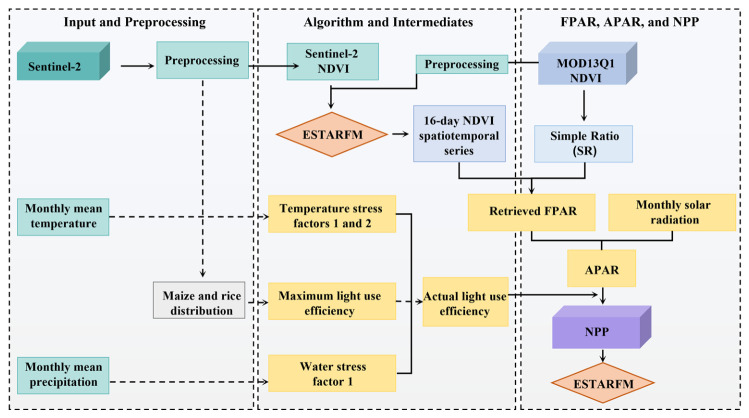
Technical workflow of the study.

This study aims to improve the accuracy of crop NPP estimation by enhancing the retrieval accuracy of actual light-use efficiency and key Absorbed Photosynthetically Active Radiation (APAR) related parameters in the CASA model. The overall technical workflow is shown in Figure 2. First, to improve the estimation accuracy of APAR, NDVI derived from Sentinel-2 imagery and the MODIS NDVI product were used as the basic remote sensing data, and data fusion was performed using the ESTARFM method to construct an NDVI series with a temporal resolution of 16 days for the main crop growing season from June to October. Furthermore, the simple ratio (SR) was introduced, and FPAR was calculated from the fused NDVI and SR using an equal-weighting scheme to improve the retrieval accuracy of FPAR. Meanwhile, to improve the estimation accuracy of actual light-use efficiency in the CASA model, supervised classification of the spatial distributions of maize and rice was conducted based on Sentinel-2 imagery using artificial neural network (ANN), random forest (RF), support vector machine (SVM), and extreme gradient boosting (XGBoost). The model with the best overall performance was selected for crop type extraction, and the corresponding maximum light-use efficiency values were then assigned according to the extracted crop types. Subsequently, the temperature stress factors (T1 and T2) and water stress factor (W1) were calculated from monthly mean temperature and monthly precipitation, respectively, and were combined with the maximum light-use efficiency ($\varepsilon_{max}$) determined by crop type to derive the actual light-use efficiency of crops. Finally, based on the improved actual light-use efficiency and APAR, the monthly NPP time series and cumulative NPP over the entire growing season were estimated separately for maize and rice, and the model results were validated and evaluated using measured NPP data from field sampling sites.

#### 2.3.1. Vegetation Index Extraction

To characterize vegetation growth conditions in the study area, the normalized NDVI and SR were extracted from Sentinel-2 imagery. The vegetation indices were calculated using the Band Math tool in ENVI 5.6 software (L3Harris Geospatial, Broomfield, CO, USA), in which the red band was represented by Sentinel-2 Band 4 (*B*4) and the near-infrared (NIR) band by Band 8 (*B*8). *NDVI* and *SR* were then calculated from the reflectance information of the red and near-infrared bands to characterize vegetation cover conditions and growth vigor [25,26]. The equations for *NDVI* and *SR* are given as follows:

(1) $$NDVI=\frac{B8-B4}{B8+B4}$$

(2) $$SR=\frac{B8}{B4}$$

Note: In Equations (1) and (2), *B*4 and *B*8 denote the Sentinel-2 bands listed in Table 1.

#### 2.3.2. NDVI Fusion Method Based on ESTARFM

To obtain a continuous NDVI series with both high spatial and high temporal resolution, this study employed the ESTARFM to perform spatiotemporal fusion of Sentinel-2 NDVI and MODIS NDVI products. Previous studies have shown that, compared with the classical STARFM, ESTARFM exhibits more stable fusion performance in regions with complex land-cover composition and strong spatial heterogeneity, while better preserving spatial details of ground features [19,27]. By introducing multi-temporal constraints and a similar-pixel selection mechanism, this model enhances the reconstruction capability of high-resolution information in heterogeneous surface environments [4]. Within a local moving window, the high-resolution NDVI at the target date $t_{2}$ can be expressed as follows:

(3) $$F\left(x_{0},y_{0},t_{2},B\right)=F\left(x_{0},y_{0},t_{1},B\right)+\sum_{i=1}^{N}W_{i}V_{i}\left[C\left(x_{i},y_{i},t_{2},B\right)-C\left(x_{i},y_{i},t_{1},B\right)\right]$$

where $F\left(x_{0},y_{0},t_{2},B\right)$ denotes the high-spatial-resolution NDVI value of the predicted pixel at location $\left(x_{0},y_{0}\right)$ on the target date $t_{2}$; $F\left(x_{0},y_{0},t_{1},B\right)$ denotes the high-spatial-resolution NDVI value of the central pixel at the same location on the reference date $t_{1}$; $C\left(x_{i},y_{i},t_{2},B\right)$ and $C\left(x_{i},y_{i},t_{1},B\right)$ denote the low-spatial-resolution NDVI values of the ith pixel within the moving window on dates $t_{2}$ and $t_{1}$, respectively; $W_{i}$ represents the weight of the ith similar pixel; $V_{i}$ is the conversion coefficient derived from mixed-pixel decomposition; N is the total number of similar pixels involved in the prediction within the moving window; $\left(x_{0},y_{0}\right)$ denotes the spatial coordinates of the central pixel to be predicted; $\left(x_{i},y_{i}\right)$ denotes the spatial coordinates of the ith similar pixel within the window; $t_{1}$ is the reference date; $t_{2}$ is the target prediction date; and B denotes the corresponding NDVI data.

#### 2.3.3. Crop Spatial Distribution Mapping

To accurately obtain the spatial distribution of crop types in the study area, four supervised learning algorithms—ANN, SVM, XGBoost, and RF—were selected for comparative crop classification. These algorithms represent neural network-based methods, kernel-based methods, and ensemble learning approaches, including both boosting- and bagging-based models. This design enables a comprehensive comparison of their performance in classifying maize and rice in the study area from different modeling perspectives, thereby allowing the optimal model to be selected for extracting the spatial distribution of crop types.

First, classification features were constructed based on the preprocessed remote sensing imagery. Second, labeled samples of maize, rice, and other land-cover types were obtained from sample points or sample plots and divided into a training set and a validation set at a ratio of 8:2 using stratified random sampling, with the training set used for model training and the validation set used for classification accuracy assessment. Subsequently, ANN, SVM, XGBoost, and RF were separately employed to automatically classify crop types in the study area. Finally, overall accuracy (OA) and the Kappa coefficient were calculated based on the validation set to comprehensively evaluate the classification results of different models, and the model with the highest accuracy was selected as the final classification model [28,29,30].

Among these models, ANN learns the complex relationships between input features and class labels through multilayer nonlinear mapping [31]. SVM performs sample classification by constructing an optimal separating hyperplane and can handle nonlinear problems through the use of kernel functions [32]. XGBoost is an efficient tree-based ensemble method based on gradient boosting. It fits residuals iteratively in a stage-wise manner and incorporates regularization to reduce the risk of overfitting, thereby achieving both high training efficiency and strong predictive performance [33,34]. RF consists of multiple decision trees and employs bootstrap resampling together with random feature subset selection for bagging-based ensemble learning. This approach can effectively reduce model variance, improve robustness to noise, and provide measures of variable importance to support feature interpretation [35,36].

#### 2.3.4. APAR Retrieval Method Based on NDVI and SR

This study is based on the CASA model and mainly improves the model from the perspective of FPAR retrieval to enhance the accuracy of NPP estimation in agricultural areas. The estimation of NPP in the CASA model is based on the principles of vegetation photosynthesis [11,37,38], and its core formulation is shown in Equations (4) and (5).

(4) $$NPP\left(x,t\right)=APAR\left(x,t\right)\times LUE\left(x,t\right)$$

(5) $$APAR\left(x,t\right)=PAR\left(x,t\right)\times FPAR\left(x,t\right)$$

where NPP$\left(x,t\right)$ denotes the net primary productivity fixed by vegetation for pixel x in month t with a unit of $gC\cdot m^{-2}\cdot {month}^{-1}$; $APAR\left(x,t\right)$ denotes the absorbed photosynthetically active radiation of pixel x in month t with a unit of $MJ\cdot m^{-2}\cdot {month}^{-1}$; $PAR\left(x,t\right)$ denotes the incident photosynthetically active radiation of pixel x in month t, with a unit of $MJ\cdot m^{-2}\cdot {month}^{-1}$; and $LUE\left(x,t\right)$ denotes the actual light-use efficiency of pixel x in month t, with a unit of $gC\cdot {MJ}^{-1}$. A large number of previous studies have demonstrated that $PAR\left(x,t\right)$ is generally approximately 50% of $SOL\left(x,t\right)$ [11]. $FPAR\left(x,t\right)$ denotes the fraction of photosynthetically active radiation absorbed by pixel x in month t, which is mainly regulated by vegetation physiological parameters such as leaf area index. In general, vegetation with a larger leaf area tends to exhibit a higher FPAR because its canopy can intercept more photosynthetically active radiation. In this study, to reduce the uncertainty introduced by cloud cover in the calculation of vegetation indices from remote sensing imagery, a high-temporal-resolution NDVI time series was used to estimate FPAR. Previous studies have shown that NDVI can reflect vegetation growth conditions and is linearly related to FPAR [39,40]. Therefore, the maximum values of NDVI and FPAR can be determined from remote sensing imagery to further calculate $FPAR\left(x,t\right)$. Previous studies have indicated that FPAR calculated based on NDVI tends to be overestimated, whereas FPAR derived from SR is generally underestimated [41]. Therefore, using the mean of ${FPAR}_{NDVI}$ and ${FPAR}_{SR}$ can improve the robustness of FPAR estimation to a certain extent [42]. The specific equations are as follows:

(6) $$FPAR\left(x,t\right)=0.5\times \left({FPAR}_{NDVI}+{FPAR}_{SR}\right)$$

(7) $${FPAR}_{NDVI}=\frac{\left[NDVI\left(x,t\right)-{NDVI}_{min}\right]\left({FPAR}_{max}-{FPAR}_{min}\right)}{\left({NDVI}_{max}-{NDVI}_{min}\right)}+{FPAR}_{min}$$

(8) $${FPAR}_{SR}=\frac{\left[SR\left(x,t\right)-{SR}_{min}\right]\left({FPAR}_{max}-{FPAR}_{min}\right)}{\left({SR}_{max}-{SR}_{min}\right)}+{FPAR}_{min}$$

where ${FPAR}_{max}$ = 0.950 and ${FPAR}_{min}$ = 0.001 are constants, where ${NDVI}_{max}$ and ${NDVI}_{min}$ represent the maximum and minimum NDVI values in the study area for the corresponding month, respectively; ${SR}_{max}$ and ${SR}_{min}$ represent the maximum and minimum SR values in the study area for the corresponding month, respectively; and ${FPAR}_{NDVI}$ and ${FPAR}_{SR}$ values derived from NDVI and SR, respectively.

#### 2.3.5. Calculation of Actual Light-Use Efficiency Based on the Distribution of Rice and Maize

According to the calculation principle of actual light-use efficiency in the CASA model [11,43,44], and with reference to the parameterization methods for maximum light-use efficiency of crops reported in previous studies, the actual light-use efficiency of rice and maize in the study area was calculated in this study. The specific equations are as follows [11,43,44]:

(9) $$LUE\left(x,t\right)=T_{\varepsilon 1}\left(x,t\right)\times T_{\varepsilon 2}\left(x,t\right)\times W_{\varepsilon }\left(x,t\right)\times \varepsilon_{max}$$

where $LUE\left(x,t\right)$ denotes the actual light-use efficiency of pixel x in month t, with a unit of $gC\cdot {MJ}^{-1}$; $T_{\varepsilon 1}\left(x,t\right),T_{\varepsilon 2}\left(x,t\right)$, and $W_{\varepsilon }\left(x,t\right)$ denote the low-temperature stress factor, high-temperature stress factor, and water stress factor, respectively, which characterize the effects of temperature and water conditions on vegetation light-use efficiency; and $\varepsilon_{max}$ denotes the maximum light-use efficiency that vegetation can achieve under ideal environmental conditions. Previous studies have shown that $\varepsilon_{max}$ differs among crop types because of differences in canopy structure, growth duration, and photosynthetic characteristics [16,44]. Therefore, crop-specific $\varepsilon_{max}$ values were adopted in this study. Based on previous CASA-related studies and crop-specific parameter settings [11,43,44], the $\varepsilon_{max}$ values for rice and maize were set to 0.9 and 1.1 [44], respectively. Although these literature-based values provide a practical basis for CASA parameterization, their potential uncertainty is acknowledged and further discussed in Section 4.

(10) $$T_{\varepsilon 1}\left(x,t\right)=0.8+0.02\times T_{opt}\left(x,t\right)-0.0005\times {\left(T_{opt}\left(x,t\right)\right)}^{2}$$

(11) $$T_{\varepsilon_{2}}\left(x,t\right)=\frac{1.184}{1+\exp\left[0.2\times \left(T_{\mathrm{opt}}\left(x\right)-10-T\left(x,t\right)\right)\right]}\times \frac{1}{1+\exp\left[0.3\times \left(-T_{\mathrm{opt}}\left(x\right)-10+T\left(x,t\right)\right)\right]}$$

(12) $$W_{\varepsilon }\left(x,t\right)=0.5+0.5\times \frac{E\left(x,t\right)}{P\left(x,t\right)}$$

Here, $T_{\varepsilon 1}\left(x,t\right)$ and $T_{\varepsilon 2}\left(x,t\right)$ represent the low-temperature stress factor and high-temperature stress factor of pixel x in month t, respectively, and are used to characterize the inhibitory effects of temperature conditions on vegetation light-use efficiency. $W_{\varepsilon }\left(x,t\right)$ represents the water stress factor of pixel x in month t and reflects the influence of water conditions on vegetation light-use efficiency. $T_{opt}\left(x\right)$ denotes the optimum temperature for vegetation growth and is defined as the monthly mean temperature corresponding to the month in which NDVI reaches its peak during the entire growing cycle of vegetation within pixel xxx. $T\left(x,t\right)$ denotes the mean temperature of pixel x in month t. $E\left(x,t\right)$ and $P\left(x,t\right)$ denote the actual evapotranspiration and potential evapotranspiration of pixel x in month *t*, respectively, and both are typically expressed in mm.

Therefore, temperature stress factors, the water stress factor, FPAR, and total solar radiation jointly influence the spatiotemporal distribution of NPP in the CASA model.

#### 2.3.6. Evaluation Methods

To quantitatively verify the reliability of the improved model estimation results, four widely used statistical metrics were adopted to evaluate the model accuracy, including *R*^2^, root mean square error (RMSE), MAE, and MAPE. The specific calculation equations and corresponding definitions of each metric are given as follows.

$R^{2}$ is used to measure the ability of the predicted values to explain the variation in the reference values, that is, the extent to which the model can reproduce the variation characteristics of the reference values. The equation is given as follows:

(13) $$R^{2}=1-\frac{\sum_{i=1}^{n}{\left(y_{i}-{\hat{y}}_{i}\right)}^{2}}{\sum_{i=1}^{n}{\left(y_{i}-\hat{y}\right)}^{2}}$$

where $y_{i}$ is the observed value of the *i*th sample, $\hat{y_{i}}$ is the corresponding model-predicted value, $\hat{y_{i}}$ is the mean of the observed values, and n is the number of samples. In general, an $R^{2}$ value closer to 1 indicates a higher degree of agreement between the predicted and reference values.

The MAE is used to measure the average magnitude of the absolute errors between the predicted values and the observed values. The equation is given as follows:

(14) $$MAE=\frac{1}{n}\sum_{i=1}^{n}\left|y_{i}-{\hat{y}}_{i}\right|$$

where $\left|y_{i}-{\hat{y}}_{i}\right|$ represents the absolute error of an individual sample. The unit of MAE is the same as that of the original variable. Therefore, a smaller MAE indicates a lower average deviation and higher prediction accuracy.

The MAPE is used to reflect the relative error level of model predictions by emphasizing the magnitude of the error relative to the reference value, thereby facilitating comparisons among datasets with different magnitudes. The equation is given as follows:

(15) $$MAPE=\frac{1}{n}\sum_{i=1}^{n}\left|\frac{y_{i}-{\hat{y}}_{i}}{y_{i}}\right|$$

The MAPE is usually expressed as a percentage, and a smaller value indicates a lower relative error in model prediction. This metric can more intuitively reflect the deviation between the predicted values and the reference values.

The RMSE is used to measure the overall magnitude of the errors between the predicted values and the observed values, and it is more sensitive to larger errors. The equation is given as follows:

(16) $$RMSE=\sqrt{\frac{1}{n}\sum_{i=1}^{n}{\left(y_{i}-\hat{y_{i}}\right)}^{2}}$$

where *n* is the number of samples, $y_{i}$ is the observed value of the ith sample, and $\hat{y_{i}}$ is the corresponding model-predicted value. RMSE reflects the overall magnitude of the errors between the predicted and observed values and is more sensitive to larger errors.

## 3. Results

### 3.1. Classification of Rice and Maize Planting Areas

**Figure 3:**
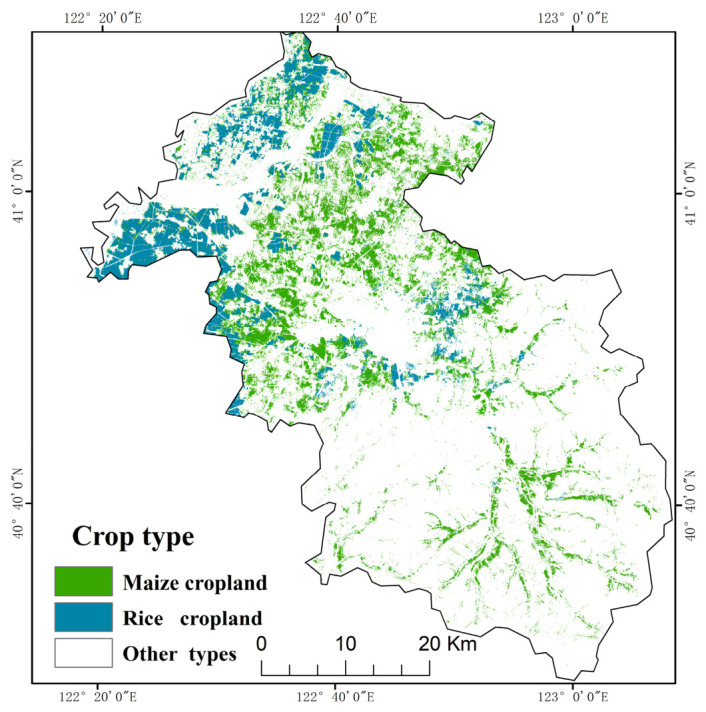
Spatial distribution of classified rice and maize.

To determine the planting distribution of rice and maize in the study area, supervised classification methods, including ANN, RF, SVM, and XGBoost, were employed to identify crop types. Among these methods, RF has a strong capability to characterize high-dimensional features and nonlinear relationships, while also showing considerable robustness to noise and multicollinearity. XGBoost exhibits excellent classification performance and anti-overfitting ability through an integrated gradient boosting framework, and is widely used in remote sensing image classification tasks. SVM generally exhibits good generalization ability under small-sample conditions, while ANN is suitable for fitting complex nonlinear relationships with its flexible network structure. The results showed that SVM performed best in crop identification in the study area and was able to effectively distinguish the spectral characteristics of rice and maize. Therefore, SVM was selected as the primary method for crop classification in the study area. The SVM classification achieved an overall accuracy (OA) of 91.8% and a Kappa coefficient of 0.89, indicating that the classification results were highly reliable and could provide robust data support for subsequent NPP estimation. The classification results are listed in Table 2 and shown in Figure 3.

### 3.2. High-Temporal-Resolution NDVI Fusion Results

**Figure 4:**
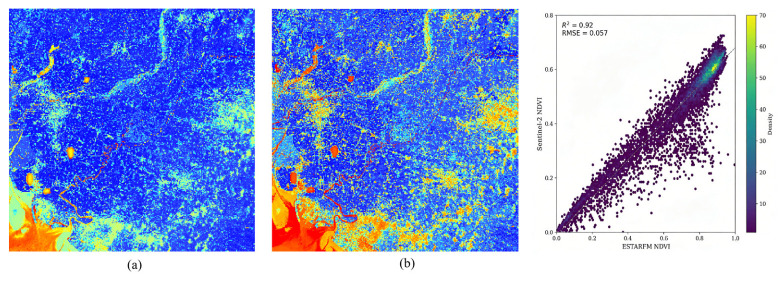
Validation results for the corresponding sampling-date imagery (**a**) Spatial distribution of reference NDVI; (**b**) Spatial distribution of fused NDVI.

The fusion results generated using the ESTARFM algorithm effectively preserved the spatial detail of the Sentinel-2 data while fully exploiting the high temporal resolution of the MODIS data. This approach alleviated the problem of insufficient temporal continuity in high-resolution NDVI data under cloudy conditions, thereby providing stable input for subsequent net primary productivity estimation. To evaluate the fusion performance of ESTARFM in the study area, more than 10,000 validation points were randomly selected within the study area using ArcGIS Desktop 10.8 (Esri, Redlands, CA, USA), and the fused results were compared with the reference data. Considering that field sample collection was mainly concentrated during the harvest period in October, Figure 4 presents the spatial distribution patterns and scatterplot-based validation results for the corresponding sampling period. Specifically, Figure 4a shows the spatial distribution of the reference NDVI, whereas Figure 4b shows the spatial distribution of the fused NDVI. Overall, the fused NDVI not only preserved the general spatial distribution pattern but also effectively recovered the spatial details of ground features. The scatter density plot shows that most sample points are distributed near the 1:1 line, indicating a high degree of consistency between the fused results and the reference data. Further quantitative analysis showed that, for this time period, the fused results achieved an $R^{2}$ of 0.92 and RMSE of 0.057, indicating that ESTARFM was able to reconstruct NDVI data with high spatial and temporal resolution for the study area with good accuracy. The accuracy assessment results for the remaining time periods are presented in Table 3.

### 3.3. Spatial Distribution of Improved FPAR

To improve the crop-specificity and accuracy of FPAR estimation, the maximum and minimum values of NDVI and SR for different crops during the main growing season in the study area were first determined before calculating FPAR based on NDVI and SR, and these values were then used as the basis for subsequent calculations. The maximum and minimum values of NDVI and SR for maize and rice during the main growing season are listed in Table 4.

**Figure 5:**
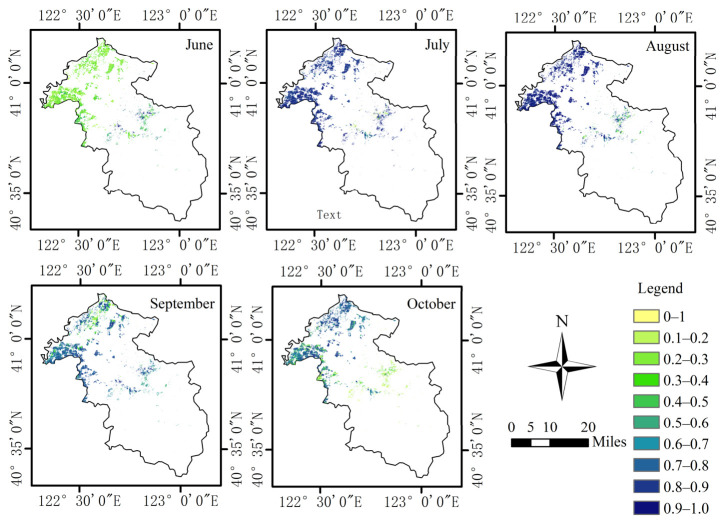
Temporal variation of rice FPAR in the study area from June to October 2025.

Figure 5 shows the seasonal variation in rice FPAR in the study area from June to October 2025. The results showed that rice FPAR exhibited an overall trend of first increasing and then decreasing throughout the growing season. In June, the mean FPAR was approximately 0.25, and the map was dominated by light green and green areas, indicating that rice canopy cover was relatively low during this period. According to the phenological characteristics of rice, May to June generally corresponded to the transplanting and regreening stages; therefore, FPAR remained at a relatively low level. In July and August, FPAR increased markedly, with blue areas gradually expanding, and the mean values rose to 0.73 and 0.79, respectively, indicating that rice had entered the vigorous growth stage and that canopy development had accelerated. In September, FPAR increased further and reached a peak value of approximately 0.86. By October, however, it declined markedly to 0.56, accompanied by a substantial reduction in high-value blue areas, suggesting that rice had entered the late growth, maturity, and harvest stages, during which canopy activity gradually weakened. Overall, rice FPAR effectively reflected the stage-specific changes in canopy development during the growing season [45].

**Figure 6:**
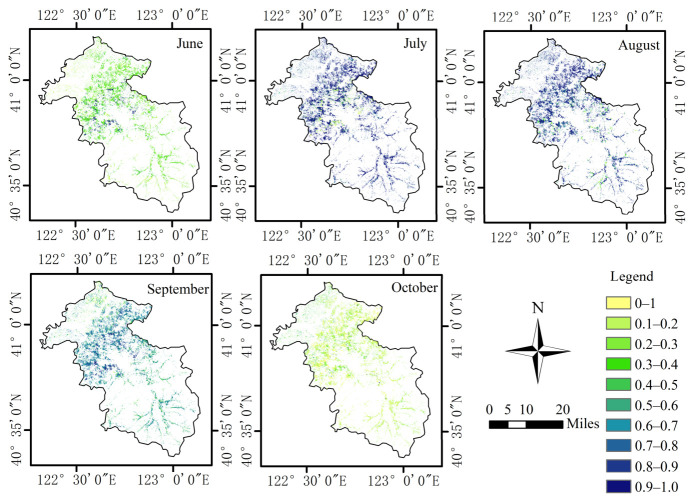
Temporal variation of maize FPAR in the study area from June to October 2025.

Figure 6 shows the seasonal variation in maize FPAR in the study area from June to October 2025. Similar to rice, maize FPAR exhibited an overall trend of first increasing and then decreasing during the growing season. The monthly mean values and standard deviations of rice and maize FPAR are presented in Table 5. In June, the mean FPAR was approximately 0.33, and the map was dominated by light green and green areas, indicating that maize canopy cover remained relatively low during this period. According to the phenological characteristics of maize, this stage generally corresponded to the seedling stage to the early jointing stage, during which the capacity of vegetation to absorb photosynthetically active radiation was relatively weak. In July and August, FPAR increased rapidly, with mean values reaching 0.84 and 0.89, respectively, and blue areas increasing markedly, reflecting that maize had entered a period of rapid growth and that canopy structure had become progressively more developed. Thereafter, FPAR declined to 0.69 in September and further decreased to 0.13 in October, when the map was mainly characterized by light green and yellow areas, indicating that vegetation cover continuously decreased after maize entered the maturity and harvest stages. Overall, the seasonal variation in maize FPAR effectively reflected the stage-specific changes in canopy development and vegetation cover during the growing season.

### 3.4. NPP Results of the Improved Mode

#### 3.4.1. Spatial Distribution and Monthly Variation Characteristics of NPP

**Figure 7:**
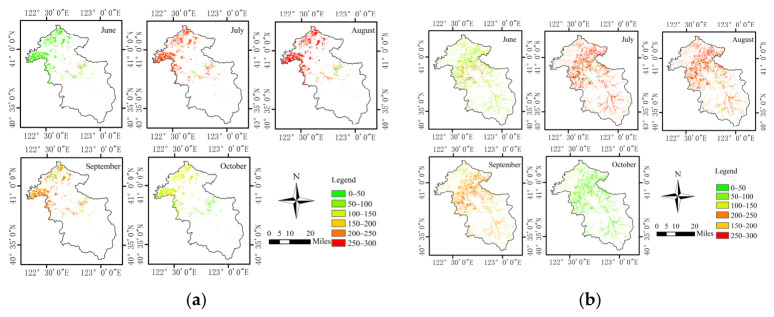
Spatial distribution of maize and rice NPP during the 2025 growing season. (**a**) Maize; (**b**) Rice.

The NPP of maize and rice estimated by the improved model exhibited pronounced stage-specific characteristics in both spatial distribution and monthly variation across the study area. As shown in Figure 7, sub-figure (a) presents the spatial distribution of maize NPP, whereas sub-figure (b) shows the spatial distribution of rice NPP during the 2025 growing season. The NPP of both crops followed a clear seasonal pattern, with relatively low values during the early growth stage, increasing values during the vigorous growth period, and declining values during the late growth stage. Specifically, low-value green areas dominated during the early growth period, followed by a gradual increase in yellow, orange, and red areas, indicating a continuous increase in NPP. During the late growth stage, however, the extent of high-value areas gradually decreased, while green and light-yellow areas increased again. High NPP values for maize mainly occurred from July to August and gradually declined after September. In contrast, rice NPP increased continuously from June onward, remained at a relatively high level from July to September, and decreased markedly in October. The monthly changes in the extent of high-value areas further indicate that crop NPP in the study area exhibited clear temporal dynamics.

**Figure 8:**
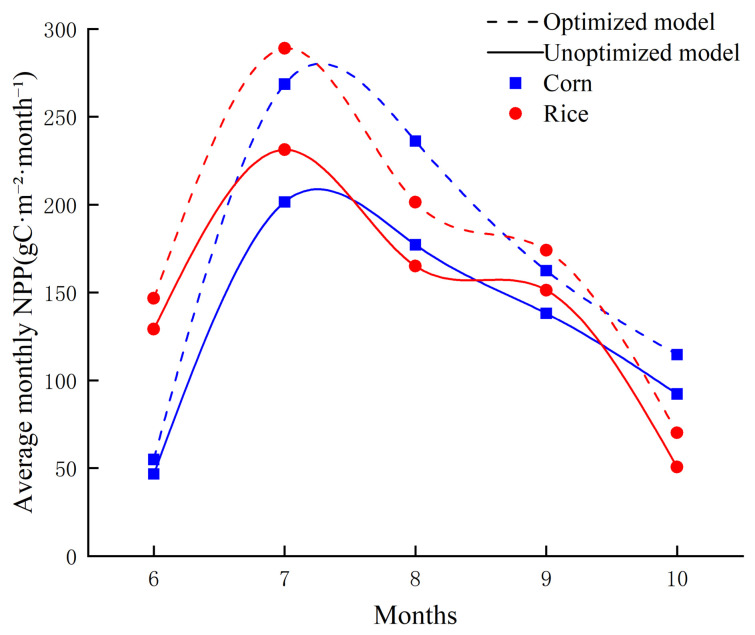
Comparison of monthly mean NPP trends of maize and rice in the study area.

The monthly mean NPP trends simulated by the optimized and unoptimized models are shown in Figure 8. For both maize and rice, monthly mean NPP first increased and then declined over the growing season. Specifically, maize reached its peak in July, whereas rice attained a relatively high level in July and remained comparatively stable from August to September. Compared with the unoptimized model, the optimized model produced slightly lower NPP estimates during the vigorous growth period, while the overall temporal variation was smoother.

#### 3.4.2. Model Accuracy Assessment

The results of field sample carbon content measurements showed that the mean carbon contents of maize and rice samples were 49.89% and 43.12%, respectively, providing the basis for calculating the measured NPP values of the crops. Further statistical analysis was conducted using the NPP data of maize and rice in the study area, and the results are presented in Table 6. Rice NPP ranged from 570.82 $\mathrm{gC}\cdot m^{-2}\cdot a^{-1}$ to 777.83 $\mathrm{gC}\cdot m^{-2}\cdot a^{-1}$, with a mean value of 676.19 $\mathrm{gC}\cdot m^{-2}\cdot a^{-1}$, whereas maize NPP ranged from 294.92 $\mathrm{gC}\cdot m^{-2}\cdot a^{-1}$ to 1280.28 $\mathrm{gC}\cdot m^{-2}\cdot a^{-1}$, with a mean value of 955.20 $\mathrm{gC}\cdot m^{-2}\cdot a^{-1}$. The wider range of maize NPP compared with that of rice indicates greater inter-field variability.

**Figure 9:**
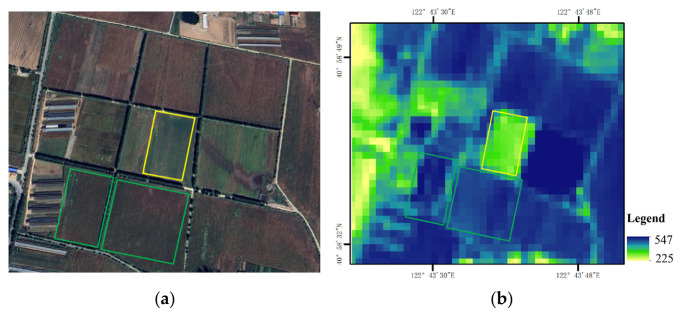
Spatial correspondence between newly planted maize fields and low-value areas in the remote sensing imagery. (**a**) Field distribution in the high-resolution imagery, where the yellow box indicates newly planted maize fields at the end of September and the green box indicates surrounding conventional maize fields; (**b**) Corresponding remote sensing imagery. The yellow-boxed area appears as a low-value region, consistent with the field survey observations and measured NPP results.

The validation results for the typical sample area further supported the above analysis. As shown in Figure 9, newly planted maize fields identified in the high-resolution imagery at the end of September corresponded to low-value areas in the remote sensing inversion results, which were consistent with the field survey observations. This indicates that the remote sensing inversion results were able to effectively reflect plot-scale differences in NPP within the study area and therefore exhibited high reliability.

The accuracy assessment results of the improved model for estimating maize and rice NPP in the study area are presented in Table 7 and Table 8. Overall, the improved CASA model showed lower estimation errors than the original CASA model for both maize and rice. For maize, the $R^{2}$ increased from 0.75 to 0.88, the MAPE decreased from 67.00% to 34.68%, and the MAE decreased from 268.5 $\mathrm{gC}\cdot m^{-2}\cdot a^{-1}$ to 144.7 $\mathrm{gC}\cdot m^{-2}\cdot a^{-1}$, indicating that model improvement substantially enhanced the accuracy of maize NPP estimation. For rice, the MAPE and MAE decreased from 78.51% and 345.1 $\mathrm{gC}\cdot m^{-2}\cdot a^{-1}$ to 23.43% and 95.6 $\mathrm{gC}\cdot m^{-2}\cdot a^{-1}$ respectively. Although the $R^{2}$ value for rice decreased slightly from 0.82 to 0.80, the error metrics improved markedly, indicating that the improved model performed better in reducing estimation bias.

The ablation experiment further demonstrated the contribution of the SR component. Compared with the full improved CASA model, the improved CASA model without SR showed higher MAPE and MAE for both maize and rice. For maize, after removing SR, MAPE and MAE increased from 34.68% and 144.7 $\mathrm{gC}\cdot m^{-2}\cdot a^{-1}$ to 45.90% and 186.4 $\mathrm{gC}\cdot m^{-2}\cdot a^{-1}$, respectively. For rice, MAPE and MAE increased from 23.43% and 95.6 $\mathrm{gC}\cdot m^{-2}\cdot a^{-1}$ to 34.60% and 142.8 $\mathrm{gC}\cdot m^{-2}\cdot a^{-1}$, respectively. These results indicate that the SR-based FPAR component contributed positively to reducing NPP estimation errors.

Overall, the improved CASA model effectively improved the estimation accuracy of maize and rice NPP in the study area, providing a reliable basis for subsequent analysis.

## 4. Discussion

In this study, the NDVI time series generated through ESTARFM fusion was integrated with the improved CASA model, thereby improving the accuracy of crop NPP estimation in the agricultural area of Haicheng. This improvement was mainly reflected in three aspects. First, the temporal continuity of vegetation index inputs under cloudy conditions was enhanced. Previous studies have shown that ESTARFM and its improved variants can effectively improve the representation of crop growth processes and phenological stages in heterogeneous agricultural areas [46]. Second, SR was incorporated together with NDVI for FPAR estimation, which helped reduce the uncertainty caused by NDVI saturation under dense crop canopy conditions and improved the reliability of APAR and NPP estimation [17]. Third, the key parameters of the CASA model were optimized according to different crop types, and crop-specific maximum light-use efficiency values were assigned for rice and maize, thereby enabling the model to represent physiological differences among crops more reasonably. Previous studies have shown that light-use efficiency (LUE) not only differs significantly among crop types but also exhibits pronounced temporal dynamics within a single growing season.

Compared with purely empirical models, the improved CASA framework has a clearer physiological basis because NPP is estimated through APAR and light-use efficiency rather than only through statistical relationships between vegetation indices and field measurements. Compared with more complex process-based crop models, this framework requires fewer field management and physiological parameters, making it more suitable for regional-scale remote sensing applications. However, the ESTARFM CASA framework still depends on the quality of optical remote sensing inputs, crop classification, meteorological data, and parameter settings. Its performance may therefore be affected in regions with persistent cloud cover, fragmented fields, complex cropping systems, or large differences in irrigation and fertilization management.

The seasonal variations in FPAR and NPP further indicate that rice and maize exhibit distinct temporal patterns during the growing season. Specifically, high values for rice were mainly maintained from July to September, whereas high values for maize were concentrated from July to August, which is consistent with the differences between the two crops in phenological characteristics and growth duration. Rice generally maintains relatively high canopy activity during the heading and grain filling stages, whereas maize reaches its peak canopy development earlier and then declines more rapidly during the late growing stage. Previous studies have shown that different crops usually differ significantly in the shape of their temporal trajectories, the timing of peak values, and the rhythm of senescence [46,47]. The spatial distribution of NPP in different months, together with the validation results for typical areas, further demonstrates that the improved model can not only effectively characterize the stage-specific changes in crop carbon accumulation, but also identify inter-field differences within the region, indicating good regional applicability and strong plot-scale representation capability.

It should be noted that this study still involves certain uncertainties. First, the number of sample points used for accuracy validation was relatively limited, and field sampling was mainly conducted during the late growing season. Therefore, the validation results should be interpreted as evidence at the site level for seasonal accumulated NPP rather than a complete representation of all phenological stages. Although public NPP products can provide useful external references, their relatively coarse spatial resolution and lack of crop-specific information may limit their direct use for validating 10 m maize and rice NPP estimates. Future studies should increase the number of field samples, collect observations across multiple key growth stages, and incorporate more suitable independent datasets to build a more comprehensive validation dataset. Second, uncertainty remains in the parameterization of maximum light-use efficiency. Although crop-specific maximum light-use efficiency values were assigned for maize and rice in this study, these values were not locally calibrated using long-term flux observations or biomass measurements across multiple growth stages. In practice, maximum light-use efficiency may be affected by cultivar differences, planting density, fertilization, irrigation, field management practices, and environmental stresses [48]. Therefore, future studies should conduct sensitivity analysis and local calibration of this parameter to further improve the robustness of CASA-based crop NPP estimation. Third, the NPP estimation process involves multiple components, including NDVI fusion, crop classification, meteorological forcing, FPAR estimation, and the parameterization of maximum light-use efficiency. Errors arising from any of these components may propagate to the final results [43]. In particular, uncertainty in the fused NDVI series during cloudy periods and potential classification errors near field boundaries may affect the spatial distribution of NPP. Differences in harvest timing, newly planted fields, and variations in field management may further increase the uncertainty of the spatial distribution of NPP during the late growing stage. Therefore, future studies should further optimize the model by incorporating observations over multiple years and growth stages, and the proposed framework should be further tested across different regions, crop types, and cropping systems to evaluate its applicability and stability under different environmental and management conditions.

This study demonstrates that integrating spatiotemporal fusion techniques with the improved CASA model is an effective approach for improving the accuracy of remote sensing-based crop NPP estimation in cloudy regions. This method is not only suitable for monthly NPP mapping in the study area, but can also provide technical support for crop carbon sequestration assessment and regional agroecological management.

## 5. Conclusions

In this study, Sentinel-2 and MODIS NDVI data were fused using ESTARFM, and the NPP of maize and rice in the agricultural area of Haicheng City was estimated using the improved CASA model. The results demonstrated that the fused NDVI time series with high spatial and temporal resolution effectively improved the continuity of crop growth monitoring under cloudy conditions. In addition, the improved CASA model better captured the spatiotemporal variation in maize and rice NPP and enhanced the accuracy of NPP estimation. Overall, this approach provides technical support for carbon sequestration assessment in agroecosystems and agricultural ecological management. Future studies should focus on the localized optimization of light-use efficiency parameters for major crops in Northeast China and on the integration of more efficient spatiotemporal fusion methods to further improve the applicability and estimation accuracy of the model under different regional and crop conditions.

## Figures and Tables

**Table 1:** Parameters of Sentinel-2 spectral bands.

	Band	Wavelength (nm)	Description	Resolution (m)
MSI	Band1	443	Coastal aerosol	60
	Band2	490	Blue	10
	Band3	560	Green	10
	Band4	665	Red	10
	Band5	705	Vegetation red edge	20
	Badn6	740	Vegetation red edge	20
MSI	Band7	783	Vegetation red edge	20
	Band8	842	NIR	10
	Band8A	865	Narrow NIR	20
	Band9	945	Water vapor	60
	Band10	1375	SWIR–cirrus	20
	Band11	1610	SWIR-1	20
	Band12	2190	SWIR-2	20

**Table 2:** Comparison of classification accuracy among different models.

Model	OA (%)	Kappa
ANN	86.5	0.81
SVM	91.8	0.89
XGBoost	90.6	0.88
RF	90.9	0.88

**Table 3:** Accuracy assessment results of ESTARFM-fused NDVI for different time periods.

Date	Maize Phenological Stage	Rice Phenological Stage	$R^{2}$	RMSE
21 June 2025	Jointing stage	Tillering stage	0.82	0.073
11 July 2025	Tasseling stage	Booting stage	0.94	0.037
19 October 2025	Harvest stage	harvest stage	0.92	0.057

**Table 4:** NDVI and SR values during the main growing season (June–October).

Crop	Index	Statistic	Jun	Jul	Aug	Sep	Oct
Maize	NDVI	Max	0.735	0.917	0.852	0.791	0.221
		Min	0.327	0.834	0.831	0.675	0.200
	SR	Max	6.547	23.096	12.514	8.569	1.567
		Min	1.972	11.048	10.834	5.154	1.500
Rice	NDVI	Max	0.653	0.881	0.831	0.656	0.489
		Min	0.417	0.714	0.740	0.560	0.427
	SR	Max	4.764	15.807	10.834	4.814	2.914
		Min	2.431	5.993	6.692	3.545	2.490

**Table 5:** Monthly mean values and standard deviations of rice and maize FPAR.

Month	Rice Mean	Rice SD	Maize Mean	Maize SD
6	0.25	0.12	0.33	0.14
7	0.73	0.07	0.87	0.06
8	0.79	0.07	0.82	0.08
9	0.86	0.18	0.69	0.14
10	0.56	0.21	0.13	0.17

Note: Standard deviation was used to describe the variability of FPAR among different fields within the study area.

**Table 6:** Statistical summary of measured NPP for maize and rice in the study area.

Crop	Minimum	Maximum	Mean
Maize	294.92	1280.28	955.20
Rice	570.82	777.83	676.19

Unit: gC·m^−2^·yr^−1^.

**Table 7:** Comparison of NPP estimation accuracy for maize and rice among different models.

Crop	Model	$R^{2}$	MAPE (%)	MAE (gC·m^−2^·a^−1^)
Maize	Improved CASA	0.88	34.68	144.7
	Improved CASA (without SR)	0.82	45.9	186.4
	Original CASA	0.75	67.00	268.5
Rice	Improved CASA	0.80	23.43	95.6
	Improved CASA (without SR)	0.80	34.6	142.8
	Original CASA	0.82	78.51	345.1

Note: Improved CASA (without SR) denotes the improved CASA model without the SR-based FPAR component. MAPE is expressed as %, and MAE is expressed in $gC\cdot m^{-2}\cdot a^{-1}$.

**Table 8:** Comparison of NPP estimation results against ground measurements and remote sensing products.

Crop	Dataset	Original CASA			Improved CASA		
		$R^{2}$	MAPE	MAE	$R^{2}$	MAPE	MAE
Maize	Ground measurements	0.75	67.00	268.5	0.88	34.68	144.7
	Remote sensing product	0.51	85.70	350.4	0.65	74.5	185.8
Rice	Ground measurements	0.82	78.51	345.1	0.80	23.43	95.6
	Remote sensing product	0.45	98.10	365	0.58	79.8	214.7

Note: MAPE is expressed as %, and MAE is expressed in $gC\cdot m^{-2}\cdot a^{-1}$.

## Data Availability

The original contributions presented in this study are included in the article. Further inquiries can be directed to the corresponding author.
